# Explaining variation of implementation outcomes of centralized waiting lists for unattached patients: A comparative multiple case study in Quebec

**DOI:** 10.1108/JHOM-10-2018-0303

**Published:** 2019-08-08

**Authors:** Sabina Abou Malham, Mélanie-Ann Smithman, Nassera Touati, Astrid Brousselle, Christine Loignon, Carl-Ardy Dubois, Kareen Nour, Antoine Boivin, Mylaine Breton

**Affiliations:** 1 Université de Sherbrooke – Campus de Longueuil, Longueuil, Canada; 2 École nationale d’administration publique, Montréal, Canada; 3 School of Public Administration, Université de Sherbrooke – Campus de Longueuil, Longueuil, Canada; 4 University of Victoria, Victoria, Canada; 5 Département de gestion, d’évaluation et de politique de santé, Université de Montréal, Montreal, Canada; 6 Centre intégré de sante et de services sociaux de la Montérégie-Centre, Greenfield Park, Canada; 7 Département de médecine de famille et médecine d’urgence, Faculté de médecine, Université de Montréal, Montreal, Canada; 8 Canadian Research Chair in Clinical Governance on Primary Health Care, Longueuil, Canada

**Keywords:** Primary healthcare, Waiting lists, Implementation effectiveness, Family practice, Case study

## Abstract

**Purpose:**

Centralized waiting lists (CWLs) for patient attachment to a primary care provider have been implemented across Canada, including Quebec. Little is known about the implementation of CWLs and the factors that influence implementation outcomes of such primary care innovations. The purpose of this paper is to explain variations in the outcomes of implementation by analyzing the characteristics of CWLs and contextual factors that influence their implementation.

**Design/methodology/approach:**

A multiple qualitative case study was conducted. Four contrasting CWLs were purposefully selected: two relatively high-performing and two relatively low-performing cases with regard to process indicators. Data collected between 2015 and 2016 drew on three sources: 26 semi-structured interviews with key stakeholders, 22 documents and field notes. The Consolidated Framework for Implementation Research was used to identify, through a cross-case comparison of ratings, constructs that distinguish high from low-performing cases.

**Findings:**

Five constructs distinguished high from low-performing cases: three related to the inner setting: network and communications; leadership engagement; available resources; one from innovation characteristics: adaptability with regard to registration, evaluation of priority and attachment to a family physician; and, one associated with process domain: engaging. Other constructs exerted influence on implementation (e.g. outer setting, individual characteristics), but did not distinguish high and low-performing cases.

**Originality/value:**

This is the first in-depth analysis of CWL implementation. Results suggest important factors that might be useful in efforts to continuously improve implementation performance of CWLs and similar innovations.

## Background

Implementation of innovations is a key step in the diffusion–dissemination–implementation process in terms of maximizing the likelihood of achieving beneficial outcomes (Nilsen, 2015; Durlak, 2015; Damschroder *et al.*, 2009). In the health services field, implementation of tailored healthcare innovations is recognized as a critical strategy for improving health service delivery, health system performance and patient outcomes (Flottorp *et al.*, 2013; Grol and Grimshaw, 2003). However, ensuring implementation in a real-world setting and integrating the innovation into daily routine practice remains complex and challenging (Fixsen *et al.*, 2005; Fleuren *et al.*, 2004; Grol and Grimshaw, 2003). The success or failure of implementation is often associated with context (Proctor *et al.*, 2011), which encompasses, according to Pfadenhauer *et al.* (2017), “not only the physical structure but also the dynamic roles, interactions and relationships, within which the innovation unfolds and interacts (p. 6)” (Kirk *et al.*, 2016; Damschroder *et al.*, 2009; Pfadenhauer *et al.*, 2017). Moreover, contextual factors may explain variations in the way an innovation is implemented. Research investigating variations in the implementation of a particular innovation can help to understand which implementation strategy works best for which patient group, and under what conditions variations in implementation influence healthcare delivery and patient outcomes (Champagne *et al.*, 2011; Liang *et al.*, 2016; Damschroder *et al.*, 2011).

Despite increasing recognition that context must be considered when looking at variations in the effectiveness of implementation, this research domain is still evolving. Some authors (Liang *et al.*, 2016; Benzer *et al.*, 2013; Varsi and Ekstedt, 2015; Hartmann *et al.*, 2016) have stressed the need to conduct empirical studies that include multiple sites and explore how stakeholders in different organizational positions (e.g. health professionals, managers, administrative staff) perceive the implementation of an innovation, in order to identify contextual factors that distinguish high from low-performing cases (Benzer *et al.*, 2013; Varsi and Ekstedt, 2015). Indeed, identifying differences across contexts regarding how to embrace complex innovations call for multisite studies to assure replicability (Miles *et al.*, 2014).

Innovations are introduced regularly in all countries to address problems that appear in healthcare systems. In Canada, the high number of patients without a regular primary healthcare provider has gained increasing attention in reform efforts (Breton, Smithman, Brousselle, Loignon, Touati, Dubois, Nour, Boivin, Berbiche and Roberge, 2017). Canada’s rate of unattached patients compares poorly to other countries in the Organisation for Economic Co-operation and Development, such as France, Norway and Germany, where under 5 percent of the population reports lacking a regular primary care provider (Statistics Canada, 2014). With approximately 15 percent of the population reporting not having a regular primary care provider, Canada ranks only somewhat better than the USA (23 percent of population unattached) and the UK (19 percent) (Statistics Canada, 2014; Breton, Smithman, Brousselle, Loignon, Touati, Dubois, Nour, Boivin, Berbiche and Roberge, 2017). Canadian provinces range from 25 percent in Quebec to 8 percent in Ontario. To confront this problem, seven provinces have implemented an innovative organizational model, creating centralized waiting lists (CWLs) for unattached patients in primary healthcare (Breton, Green, Kreindler, Sutherland, Jbilou, Wong, Shaw, Crooks, Contandriopoulos, Smithman and Brousselle, 2017). CWLs are used to centralize requests for family physicians in a given territory, and match patients with physicians according to urgency of medical needs and availability of primary care providers (Breton, Gagne and Gankpé, 2014; Breton, Green, Kreindler, Sutherland, Jbilou, Wong, Shaw, Crooks, Contandriopoulos, Smithman and Brousselle, 2017). CWLs have been implemented in many fields of healthcare, particularly as a way to manage waiting lists for elective surgery (McGurran and Noseworthy, 2002). To our knowledge, CWLs have not been used outside Canada to match primary healthcare providers with unattached patients. Moreover, these complex models in primary care implementation are unexplored. Questions remain regarding what differentiates high from low-performing CWLs, and what contextual (e.g. social, political, geographical factors), and organizational factors (e.g. culture, climate for change), and characteristics of both the innovation (e.g. relative advantage, adaptability) and the individual (e.g. knowledge, attitudes) are associated with the effectiveness of implementation (Chaudoir *et al.*, 2013; Damschroder *et al.*, 2009, 2011). Indeed, with regard to improving the effectiveness of those innovations, the gap is greatest in the literature that opens up the black box and investigates contextual conditions involved in implementation success or failure (Breton, Smithman, Brousselle, Loignon, Touati, Dubois, Nour, Boivin, Berbiche and Roberge, 2017). The present paper attempts to address this gap in the implementation research.

### Study aim

This study aims to explain and understand variations in the outcomes of implementation by analyzing the characteristics of CWLs and contextual factors that influence their implementation.

### Study setting

In Canada, seven provinces have implemented CWLs to better manage supply and demand for attachment to a primary healthcare provider (Breton, Green, Kreindler, Sutherland, Jbilou, Wong, Shaw, Crooks, Contandriopoulos, Smithman and Brousselle, 2017). Although significant efforts have been invested to improve patient’s attachment access through this innovation, very few empirical studies have been conducted on CWLs implementation across Canada (Breton, Smithman, Brousselle, Loignon, Touati, Dubois, Nour, Boivin, Berbiche and Roberge, 2017).

This study analyzes CWLs implemented in the province of Quebec, considered a pioneer in CWLs for unattached patients (Breton, Brousselle, Boivin, Loignon, Touati, Dubois, Nour, Berbiche and Roberge, 2014). This province of 8m residents has attached more patients through CWLs – over 1m – with a primary care physician, than any other in Canada. Quebec has a tax-based system that provides universal access to medical services (Breton, Brousselle, Boivin, Loignon, Touati, Dubois, Nour, Berbiche and Roberge, 2014). The healthcare structure is based on three levels of governance: provincial, regional and local. At the local level, 94 Health and Social Services Centers (HSSCs) are responsible for meeting population needs, and particularly the needs of the most vulnerable, on their local territories (Levesque *et al.*, 2010).

### Innovation

**Figure 1:**
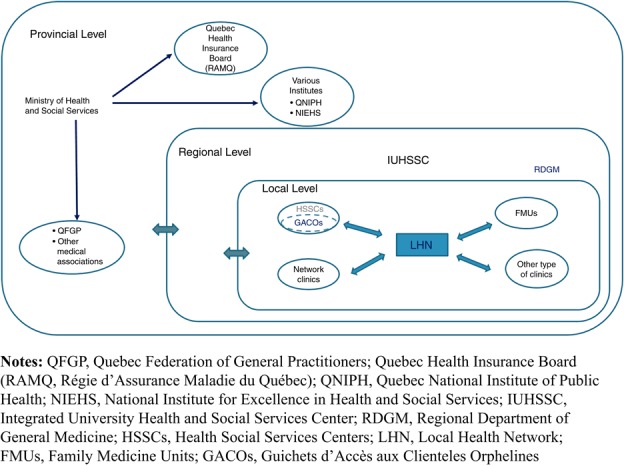
Innovation context

The CWLs in Quebec, called “Guichets d’accès aux clientèles orphelines” (GACOs) were implemented in 2008 by the Ministry of Health and Social Services (MSSS) in collaboration with the Quebec Federation of General Practitioners. They have a dual objective of: attaching patients to a family physician, and prioritizing vulnerable patients (Breton, Gagne and Gankpé, 2014). GACOs were implemented in the Local Health Network (LHN) which includes both the HSSC in which the GACO is physically located in each of the 94 HSSCs across the province, and all the primary care structures (e.g. Family Medicine Units, network clinics, etc.) included in the LHN related to that HSSC. Each HSSC, composed of several healthcare organizations, was responsible for their own GACO and had much discretion over local adoption of the innovation (see Figure 1).

**Figure 2:**
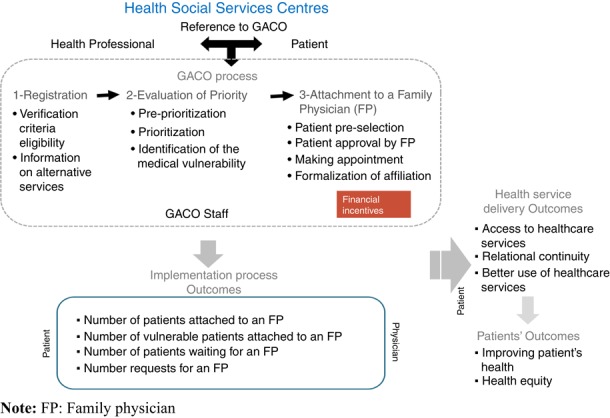
Description of the GACO model

The GACO in each HSSC is managed by a clerk who receives requests; a nurse who evaluates patient requests on a priority scale based on their clinical profile in collaboration with a local medical coordinator; a physician mandated to help attach patients with a family physician (Breton, Green, Kreindler, Sutherland, Jbilou, Wong, Shaw, Crooks, Contandriopoulos, Smithman and Brousselle, 2017). People lacking a family physician can either register directly with the GACO themselves, or be referred by a health professional (e.g. nurse, social worker, physician). Once the person is registered on the GACO, the nurse assigns a priority code based on the urgency and/or complexity of that person’s health needs. The MSSS framework recommends maximum waiting times for attachment at each of the five priority levels (1–5), ranging from less than 30 days for Priority 1 patients who require immediate medical care (e.g. complex pathologies) to no specified wait times for people in good health, considered as priority 5. Ultimately, patients are matched with a family physician based on the availability and practice characteristics of family physicians participating in the GACO, the patient’s priority category and the date of the request. Family physician participation in the GACO is voluntary and they can choose the number of patients they wish to attach from the GACO. To encourage participation, physicians receive a financial bonus for accepting an unattached patient, which is paid at the time of their first visit (Breton, Brousselle, Boivin, Loignon, Touati, Dubois, Nour, Berbiche and Roberge, 2014) (Figure 2).

Almost a decade after launching the GACO model of CWLs, a research team in Quebec published a performance assessment focused on four outcomes of the implementation process: new requests for a family physician, change in the number of patients on the waiting list and numbers of patients and vulnerable patients attached to a family physician through the CWLs (Breton, Smithman, Brousselle, Loignon, Touati, Dubois, Nour, Boivin, Berbiche and Roberge, 2017). The assessment is based on a one year-period of clinical-administrative data from the information systems of 86 of the 94 CWLs in Quebec, and shows very large performance variations between the GACOs of different regions, and even between GACOs in a same region, on all implementation process indicators (Breton, Smithman, Brousselle, Loignon, Touati, Dubois, Nour, Boivin, Berbiche and Roberge, 2017). The authors concluded that, to understand these variations, qualitative case studies were needed to compare GACOs with relatively high performance on process outcomes indicators against those with relatively weak performance.

### Conceptual framework

The Consolidated Framework for Implementation Research (CFIR) developed by Damschroder *et al.* (2009) was used in this study as it is applicable to complex implementation efforts, and comprehensively captures the interplay of factors that influence implementation of innovations in healthcare services. A meta-theoretical model based on a synthesis of 19 theories and frameworks, the CFIR is widely used in the field of implementation research (Kirk *et al.*, 2016; Varsi and Ekstedt, 2015) to understand factors underlying variation in the implementation of innovations. Studies of innovations in obesity management (Damschroder *et al.*, 2011), in health information technology (Haverhals *et al.*, 2015) and supportive housing programs for persons with serious mental illness (Gilmer *et al.*, 2013), among others, have employed this framework in their analysis. It is organized in five major domains that incorporate 39 constructs considered important to the effectiveness of implementation (Kirk *et al.*, 2016).

Taking a whole system approach, the five domains were operationalized and adapted in the present study as follows:

1. the outer setting is defined by the larger socio-demographic, economic, political, geographic context surrounding the inner setting within which the GACO is implemented (e.g. patient needs, cosmopolitanism, peer pressure, external policy, resources);
2. the inner setting refers to characteristics (e.g. structural characteristics, networks and communication, organizational culture and climate, readiness for implementation) of the organization where the GACO is implemented: the LHN. It includes both the HSSC in which the GACO is physically located, and all the primary care structures included in the LHN related to that HSSC (refer to Figure 1);
3. the characteristics of individuals involved in the implementation, including GACO staff members and the family physicians of the LHN, consider knowledge and beliefs, self-efficacy, stage of change and identification with the organization;
4. the innovation characteristics describe characteristics of the GACO related to its adaptability to a given setting, complexity, trialability, evidential support and its relative advantages (or disadvantages); and
5. the process of implementation refers to planning, engaging, executing, reflecting and evaluating the GACO process as interrelated sub-processes known to have a key role in implementation.

## Methods

### Study design

A formative evaluation using qualitative multiple case study design was chosen to gain an in-depth understanding of phenomena in “real world settings [where] the researcher does not attempt to manipulate the phenomenon of interest” (Patton, 2002).

Our study builds on results of the recent performance assessment of Quebec’s CWLs for unattached patients, which emphasized the need to better understand variations arising both from within the GACOs and from external local or regional influences (Breton, Smithman, Brousselle, Loignon, Touati, Dubois, Nour, Boivin, Berbiche and Roberge, 2017). Cases were purposefully selected by an advisory committee composed of six decision makers from the three levels of health system governance (provincial, regional and local), four healthcare professionals (two nurses and two physicians) involved in implementing and monitoring GACOs in Quebec, and four researchers from our team. Case selection was based on four performance indicators available for 2013–2014 in the CWLs’ clinical-administrative database related to the GACO implementation processes (new requests for a family physician, change in the number of patients registered with the GACO who were waiting to be assigned a family physician, and the number of patients, and vulnerable patients attached to a family physician through the GACO).

To compare performance across the province, indicators for each GACO were transformed into rates per 10,000 population, and were classified into tertiles of relative performance: low, average or high. Contrasting cases were selected (lower vs higher performing GACOs) to understand and explain contextual conditions that led to different process outcomes (Patton, 2002) and support achieving theoretical replication (Yin, 2013). Cases were chosen from within two neighboring regions, Montréal and Montérégie, the two most populous regions of Quebec (accounting together for approximately 42 percent of the province’s population), to include GACOs managing similar numbers of patients and providers. Note that none of the cases were categorized as low or high performing for all process outcomes; we selected cases based on a global assessment of performance across all indicators. For instance, our low-performing cases were classified as low for some indicators and average for others, but overall ranked among the worst GACOs in the province.

Table I presents the relative performance (implementation process outcomes) of the four selected GACOs.

### Data collection and participants

Data collection took place between May 2015 and June 2016 and drew on three main sources in order to triangulate data: semi-structured interviews with a range of GACOs stakeholders, documentation from the GACOs and field notes taken during and after interviews (Patton, 2002).

For every case, we conducted semi-structured interviews with between five and eight stakeholders with different perspectives. All staff involved in the GACO implementation (nurses, clerks, local medical coordinators and managers) and a few family physicians, with experience in attaching patients from the CWLs, were asked to take part in the study. A total of 26 key stakeholders were interviewed, using an interview guide based on the main domains of the CFIR. Interviews lasted between 45 and 90 min. It should be noted that in one case (Case 2), the local medical coordinator position was vacant at the time of data collection and the nurse declined our invitation to participate in the study.

Field notes were taken during and after each interview by the interviewers to summarize the main elements discussed with participants, to capture factors emphasized by participants, to describe observations made by interviewers, to reflect on potential explanatory factors and to note modifications to make to the interview guide or clarifications to seek in upcoming interviews. In total, 22 documents (e.g. internal communications, monitoring reports, internally developed tools) about the GACO structure and process were collected and reviewed to understand each component of the GACOs, and key stakeholder roles at strategic and operational levels.

### Analysis

All three forms of data (documents, interviews, field notes) were analyzed. Interviews were transcribed and coded using NVivo software. Documents and field notes were coded manually. Analysis followed a three-stage process. First, we applied a deductive approach using a codebook based on the CFIR to guide data coding and analysis. The codification was controlled by the technic of double coding: a research assistant and a researcher, both blinded to the implementation performance status of the four GACOs, coded all interviews independently, meeting periodically to compare and revise codes. When disagreements were observed, the codebook was adjusted and codes inspired from the CFIR were redefined to better fit the context of GACO implementation. Finally, persistent discrepancies were resolved through discussion with the larger research team. A narrative summary, based on the CFIR constructs, was created for each interview within each case to provide a rich description of each GACO’s story. A case-specific matrix with illustrative quotes for each GACO summarized information related to how each CFIR construct influenced implementation. This process led to four narrative summaries and four case-specific matrices organized according to the CFIR constructs.

Second, the first author applied ratings, based on the summaries and matrices, to the coded constructs at the GACO level, using the rating rules described by Damschroder and Lowery (2013) (see Table II for definitions of the criteria used for rating): valence (negative, positive influence on implementation, neutral effect); and, strength/magnitude (extent of discussion of each construct by study participants).

Application of the ratings was checked by two co-authors (M-AS; MB) for a random subset of constructs within each case; and a double rating was done by (M-AS) for another random subset of coded constructs.

In the third stage of analysis, we compared ratings for each construct across all four GACOs using a cross-case analytic matrix that was developed to identify patterns of variation by construct across cases. Finally, a detailed matrix of the specific CFIR constructs with a special focus on categories that distinguished between high and low implementation performance allowed us to draw conclusions on patterns of variation in factors that influenced GACO implementation outcomes. As per Damschroder and Lowery’s (2013) rules, constructs were coded as: (missing) when missing too much data to discern a pattern; (0) when data did not allow us to distinguish between high and low implementation GACOs; or (−1/+1) weakly or (−2/+2) strongly distinguishing low from high implementation performance GACOs.

In our study, we examined the difference in positive or negative ratings between high and low-performing cases to determine if the construct was an important distinguishing factor and also relied on the supporting qualitative summary. If a difference was of at least two points, the construct was considered to make a strong distinction.

To ensure scientific rigor, double blinded coding was performed on all transcripts and double rating and checks were also conducted on a random subset of constructs within each case. Also, an audit trail was kept of all changes made to the codebook as coding and analysis progressed throughout the study (Patton, 2002). Preliminary results were discussed between three authors (SAM, M-AS, MB) on several occasions early on in the study, and with the broader research team as the study advanced.

## Results

In this section, we discuss the five main constructs that helped identify relevant and rich explanations of variation between high and lower performance level cases. These constructs were: network and communications; leadership engagement; available resources; adaptability; and engaging.

Outer setting constructs (patient needs and resources; external policies and incentives); inner setting constructs (e.g. goals and feedback, relative priority, tension for change); innovation characteristics (relative advantage; complexity), and individual characteristics (knowledge attitudes and beliefs, personal attributes) exerted influence on GACO’s implementation, but did not distinguish cases by level of performance.

Tables III–VII show the constructs (valence and strength) pertaining to each domain based on the CFIR framework. Below, we discuss the most relevant results and present key illustrative quotes from the interview data. In addition, we provide one example related to constructs (inner setting) and themes distinguishing low and high-performing GACOs described in details in Table VIII.

### Inner setting

#### Networks and communication

Two levels were considered: within services of the HSSC where the GACO is implemented and between the services of the LHN.

##### Within the HSSC

There was evidence in all cases of good communication and network relationships sustained through informal and regular formal meetings of GACO staff (e.g. clerk and nurse). However, among GACO staff, collaborative practice to address common work issues and rapidly match a patient to a family physician appeared more developed in Cases 3 and 4 (rated high). In Cases 1 and 2 (rated low), staff were still trying to improve work procedures. Moreover, differences were seen between high and low performers in the extent of communication among GACO staff around the goal of minimizing delays to prioritize patients. In Cases 3 and 4 (rated high), staff had developed innovative communication strategies to solve problems arising from the complexity of the GACO process. While all sites were aware of this problem, only GACOs 3 and 4 had smoothed workflow procedures by instituting a communication system involving formal or informal procedures. In Case 4, staff recorded their initial comments and nurses and clerks developed symbol systems and codes that helped the nurse identify priority patients quickly, ensured work continuity, and enhanced patient access to a family physician:

So when patients call me back, they might tell me between noon and two o’clock. We’ve created codes so that I write that the patient called back, and can be reached between noon and two, and I put a code, for example PM. In prioritizing, the nurse will see that it’s between noon and two, with the PM code to call that person.

(Case 4 Clerk)

The most innovative practice was a formal communication strategy developed in Case 3 (rated high) using an online computer-based system to perform a quick pre-prioritization of patients with special needs and reduce delays for urgent patients (e.g. vulnerable patients) to avoid compromising their health status.

It is noteworthy that in Case 1 (rated low), despite attempts by the clerk to help the nurse prioritize participants, both manager and clerk admitted that problems persist, and said they were eager to put in place a computer communication system to facilitate pre-prioritization of patients:

We’re really waiting for the computer program that will help us prioritize them, which will help. We’d certainly like to do more to prioritize them, but we still have to take the time to assess them.

(Case 1 Manager)

##### Between services of the local health network

In Cases 3 and 4 (rated high), participants described good working relationships between GACO staff and health professionals working in collaborating clinics. In Case 3, for instance, good communication between the GACO clerk and some of the local clinics facilitated patient care in case of emergency. In Cases 3 and 4, transmission of medical information and health assessments to family physicians via the medical record of patients referred from the GACO was seen to speed follow up and increase family physician willingness to participate in the GACO.

Some participants (nurse, manager, family physician) in Case 4 (rated high) mentioned that communication and collaboration between GACO staff and physician clinics was sometimes difficult (e.g. GACO staff have to follow up with clinics to know if a patient has been refused by the physician). Nevertheless, GACO staff made efforts to organize discussion with clinics to overcome breaks in communication and minimize their negative impact on patients.

In contrast, in Case 1 (rated low), most physician clinics on the territory operated in isolation, which hampered their participation in the GACO. Poor communication between clinics limited collaborative practice and hindered family physician participation in GACO:

Here, we have many clinics, 30 or so clinics that work in silos and don’t speak to each other. There’s no real spirit of belonging to a HSSC. Now it’s starting to improve between certain clinics, but there are some who don’t participate in anything. There are some we never see. These people will almost never take on patients from the GACO.

(Case 1 Medical Coordinator)

In Case 2 (rated low), some participants reported that the GACO nurse facilitated referral of certain cases through personal contact with family physicians, and helped with patient transfers following retirement of a family physician. However, local family physicians in clinics had different perceptions and complained of poor communication from the GACO about patients on the waiting list and changes in GACO procedures. They even mentioned that the medical director of the clinic had to seek out this information himself:

Regarding patients, no, (physicians have not received information from the GACO). The […] information we received, and it’s been a while, it’s our physician lead (of the clinic) who went to get it.

(Case 2 Family Physician 1)

### Readiness for implementation

#### Leadership engagement

In Case 2 (rated low) and Cases 3 and 4 (rated high), participants emphasized the leadership of formally appointed leaders (medical coordinator) or emergent leaders (Case 4) to achieve GACO goals.

In Cases 2 and 3, medical coordinators played a role in solving problems, encouraging family physicians to attach patients, managing patient requests and attaching many vulnerable patients. In Case 4, the medical coordinator’s lack of engagement was compensated by the leadership executed by two professionals who emerged as champions and played an active role in implementation efforts: the GACO nurse, who was enthusiastic, proactive and keen on reducing long waiting times for patients, and created adaptive strategies to leverage resources; and a young family physician who was highly engaged and attached a large number of babies and vulnerable patients (400) from the GACO during his first year of practice.

Despite, similarities across Cases (2, 3, 4) regarding leadership, some key differences between low and high-performing cases were highlighted. Participants in Case 2 mentioned the difficulty of mobilizing family physicians to attach patients during a period when the medical coordinator was absent and there was a void in leadership. The manager who temporarily filled his role admitted having limited capacity to recruit family physicians and emphasized that implementation had to be physician driven to incite family physicians to attach patients from the GACO:

I went to see several clinics because I knew a few physicians who had worked here in the past. But with doctors, it takes a doctor to talk to them. I may have the best intentions, make action plans, say “I’ll go see this or that clinic.” Little things get done, but the bulk of it has to go through physicians.

(Case 2, Manager)

Also, despite the Case 2 (rated low) medical coordinator’s support for staff in resolving problems encountered with family physicians, he did not, despite intending to do so, implement a strategy to offer patients alternative health services; a more proactive approach was evident in Cases 3 and 4 (rated high). Moreover, staff expressed their concerns about lack of continuity in the medical coordinator role and difficulties sustaining improvements to the GACO after the coordinator’s departure.

In Case 1 (rated low), no specific champion was mentioned. Participants noted that the medical coordinator fulfills a traditional role, providing support when needed and dealing with problematic family physicians. Compared to other GACOs, he was insufficiently committed to enhance family physician participation. The tie with family physicians working in clinics was weak:

He (the medical director) tried to do a little public relations with the clinics. […] He doesn’t have a very aggressive management style and, as well, these are his colleagues. We can’t forget that this is a doctor talking to other doctors. It’s a delicate balance. He can’t impose on them: he’s a doctor himself. He practices in a clinic, he has his clientele, he knows what he’s talking about because he lives it every day; he can’t impose upon them […]. I don’t know what his relation is, but he’s a very nice man, not aggressive, very forthcoming, but […] that’s that.

(Case 1 Nurse)

The nurse’s statement was endorsed by the medical coordinator himself, who admitted “not twisting the arms of family physicians” to attach patients from the GACO. “No, I didn’t twist their arms like that” (Case 1 Medical coordinator). He was not known by one of the family physician interviewed practising in one clinic. “The medical coordinator at the GACO? No I don’t know who that is” (Case 1 Family Physician 2).

#### Available resources

Lack of adequate staffing, inadequate technology, high staff turnover and financial constraints were highlighted in all cases as barriers to implementing the GACOs. Inadequate human resources caused problems in both processing patients’ registration on the GACO, and in attaching them to a family physician. For example, a lack of family physician capacity on the territory had a negative impact on GACO ability to handle requests for registration. Lack of stability in the clerk’s role led to delays as new clerks had to be trained. As well, the lack of additional funds allocated for implementation efforts constrained recruitment of additional human resources. No additional staff was made available for GACO implementation efforts and no extra support was provided to the HSSC within which the GACO operated. The HSSC had to make do with existing staff, who were assigned additional tasks to implement the GACO:

That’s it? They start cutting: the nurse who was supposed to be there five days a week is now on three days. Then she leaves on retirement, and there’s a chance she won’t be replaced. I may end up on my own and then I’ll really lose my mind.

(Case 1 Clerk)

Along with human resource challenges, GACOs had difficulties accessing the medical insurance database to verify patients’ attachment status (whether they were already registered with a family physician). All cases faced these challenges, but Cases 3 and 4 (rated high) found creative ways to deal with them. Case 4 developed an adaptive strategy to optimize GACO resources by making full use of the local HSSC nursing staff. For example, a full-time clerk and several nurses who were not initially assigned to patient prioritization worked on GACO efforts whenever their schedule allowed. Training many nurses to conduct patient evaluations expanded the potential resource pool and reduced reliance on a single person:

The process works well and the fact that we’ve – I’ve talked to others about it when I find myself with other managers – trained a good number of nurses, here and there; we’re open on weekends, and sometimes people don’t show up for their Saturday or Sunday appointments. It’s a good time to catch people at home. The weekend nurses, everyone knows, turn on the computer and sign into the SIGACO (database). All that contributes to reducing the need to bring in pregnant nurses and all that. When I mention this elsewhere, nobody’s doing it […] “That’s a great idea,” they say. Because no one has a budget for this. We have a 0.8 clerk and I believe the salary of one nurse, and in some places that can be a very part-time nurse, so […] in that way, we manage to make the machine roll along smoothly.

(Case 4 Nurse)

Along the same lines, Case 3 (rate high) managed to overcome human resource constraints through their innovative pre-prioritization system. They recruited a nurse clinician working in another department who agreed to help GACO staff prioritize patients, and check their status in the medical insurance database:

I would say that she [the nurse in the other department] has a lot on her plate. She helps us depending on the number of patients she has. There are evenings when she can’t do anything for the GACO, and evenings when she can help us out […] She does some prioritization, telephone evaluations for prioritization. It depends. Sometimes we ask her to do the RAMQ [Régie d’Assurance Maladie du Quebec-Quebec Health Insurance Board] checks. Sometimes, in researching on the RAMQ site, you find that they (the patients) have found a family physician by themselves. So there’s an elimination in that time.

(Case 3 Nurse)

### Characteristics of the innovation

#### Adaptability

In all four cases, the GACO was adapted to meet community needs. Adaptations were related to one of three GACO activities: registration, evaluation of priority and attachment.

##### Registration

In response to an overwhelming volume of calls, all four cases decided to replace telephone registration by an online registration form. What differentiated high-rated and low-rated GACOs were the procedures designed to support registration of specific population groups such as newborns and homeless patients. Cases 3 and 4 (rated high) implemented procedures to facilitate registration, which was not done in low-performance cases. In Case 3, GACO staff developed an internal procedure to register newborns on the waiting list even though the provincial GACO software did not permit registration until the baby had received a health insurance number. Case 4 used an outreach strategy whereby the GACO nurse visited homeless shelters monthly to help patients sort out their eligibility for the GACO and register:

If they don’t come to the GACO, the GACO will come to them […]. So I tell them “I’m the system that comes to see you.” I’m the HSSC nurse who goes out to present them the services offered by the CLSC, and register them for the GACO as well. So then, personally giving them the leaflet. “So now you’ll get your life on track, call us back. Your wait to get a family doctor has already begun” […] I prioritize them on the spot. You check if they have their RAMQ [Quebec Health Insurance Board] number […] I look, because I have access, to see if they have a doctor. With some, I’ve even called RAMQ with them because even I get lost in the system when I call there, so you can imagine what it’s like for this poor man.

(Case 4 Nurse)

##### Evaluation of priority

A major difference between the two groups of GACOs was that Cases 3 and 4 (rated high) put in place creative initiatives to offer alternative health services while patients waited on the GACO. This was not done in lower-rated Cases 1 and 2. For instance, Case 3 offered the possibility of receiving transitional care while waiting for a regular physician from an ambulatory unit for patients with high needs. Case 4 developed a center for disease prevention for patients in good health, which helped to evaluate non-urgent patients and offer them a check-up by a nurse, based on the guidelines, while they were on the waiting list. If a change in their health status or needs was noted, the process of being attached to a family physician could be accelerated.

##### Attachment to a family physician

Cases 3 and 4 (rated high) developed strategies to deal with family physician preferences, whereas Cases 1 and 2 (rated low) complied fully with physician demands. Case 3 implemented a restrictive approach, and did not allow physician preferences to influence patient referrals. Case 4 implemented a more flexible strategy, with GACO staff adjusting referrals to physician preferences while also taking into consideration the patient’s health profile and the importance of their needs. In Case 3, GACO staff urged family physicians to attach patients referred to them, keeping an inventory of the characteristics of each family physician’s practice to minimize the chance they would refuse a patient; GACO staff would also reduce the number of referrals as needed to a given family physician to ensure that patients would be seen by them within appropriate timelines. In Case 4, despite trying to accommodate family physicians, participants decided to refer patients with mental health problems even when family physicians did not want to take them:

In general, we try to provide a mix of cases. If he just wants diabetic patients, we’ll try to maybe send him four out of five, with the fifth being a patient with diabetes and mental health issues. We put some effort into accommodating them, but also have a clear view of our reality, which is far from easy.

(Case 4 Clerk)

In Cases 3 and 4 (rated high), adaptation according to distance and region was a noteworthy theme, whereas this was not done in Cases 1 and 2 (rated low). Case 3 used postal codes to refer patients to the closest GACO; in Case 4, this was not always possible due to limited family physician capacity on the territory covered by the GACO, which did not allow patients to choose a region for attachment.

### Process

#### Engaging

The promotion of the GACO to patients and family physicians in Cases 1 and 2 (rated low) was slower and less developed than in the high-rated cases. Participants from lower performing Cases 1 and 2 acknowledged that promoting the GACO, attracting family physicians to attach patients from the GACO and encouraging patients to use GACO services, were major concerns. In interviews, participants stressed the enormous efforts required to publicize the GACO. For example, in Case 1 (rated low), they suggested promoting the GACO by distributing pamphlets to patients in clinics. In Case 2, the manager admitted that there is still a lot of work to be done with family physicians to inform them about the GACO in order to obtain results:

My own family doctor didn’t know about the GACO, and she’s on the territory. Not all small clinics attend the regional department of family physicians meetings, which keeps them up to date, so there is a need for, like in [one region], they do a lot of PR [public relations], they even have a little pamphlet, I kept it, I found it cute that the coordinator himself went around to the clinics and handed them out. Definitely, new doctors who arrive, if they’re looking […].

(Case 1 Clerk)

Compared to the other sites, Case 3 (rated high) was keen on marketing and driving use of the GACO, providing clinics with updates on adjustments made during implementation (e.g. distributing bookmarks when launching the online registration) to increase GACO use by family physicians and patients:

We promoted on-line registration at that point. We made little bookmarks that we distributed to the clinics on our territory.

(Case 3 Manager)

Case 4 (rated high) limited promotion of the GACO at first, because they were afraid of having too many patient requests, but later distributed flyers in French and English through clinics, pharmacies, and community organizations (Case 4 Clerk, Nurse Manager):

We left them in pharmacies and clinics. Little pads like this. They were all over the area.

(Case 4 Clerk)

## Discussion

Our study succeeded in identifying factors that enhance implementation effectiveness and may be used to address performance shortcomings in CWLs. Five main influencing factors were seen to operate at different levels, interact synergistically and work together in mutually reinforcing ways to produce implementation process outcomes. These factors were also seen in four other similar studies (Damschroder and Lowery, 2013; Gilmer *et al.*, 2013; Varsi and Ekstedt, 2015; Liang *et al.*, 2016) that used the CFIR to explain variation in implementation outcomes.

At the level of the inner setting, high-performing GACOs displayed greater readiness for implementation than low-performing GACOs. Consistent with three of the four similar studies (Gilmer *et al.*, 2013; Damschroder and Lowery, 2013; Liang *et al.*, 2016), we found that a key ingredient for successful implementation of healthcare innovations was the leadership engagement demonstrated by those who played an active role in supporting implementation (physician champions in our case). Only in Varsi and Ekstedt’s (2015) study did leadership not appear as an important distinguishing factor, likely due to the fact that nursing staff in all units relied on self-management rather than a unit manager. In our study, medical coordinators in the high-performing GACOs showed a high level of commitment, making connections with family physicians at different organizational levels, and positively influencing their peers in the clinics to attach vulnerable patients. They exhibited proactive leadership and responded to the needs of patients on waiting lists (e.g. by adapting interventions). Low-performing GACOs were characterized by a leadership void in one case, and a leader less enthusiastic about the GACO mission in the other.

Our results also show that in one high-performing GACO, the lack of formal medical leadership was compensated by nursing and front-line physician leaders, who emerged naturally and actively championed the innovation. These findings suggest that, involving champions based on their motivation and willingness to take an active role in the implementation process and strong belief in the cause, is an essential step (Shaw *et al.*, 2012). This should likely precede (and influence) the appointment of a champion with formal power. Our results align with suggestions by Damschroder *et al.* (2011) and Liang *et al.* (2016) that the leadership team include not only those in positions of power, but also stakeholders from different levels of the organization, who can make significant contributions to the implementation process. While local senior physicians have the authority to mobilize their peers, and are essential to implementation, operational front-line staff may also be crucial. For example, the GACO nurse who was deeply engaged in day-to-day operations exerted effective leadership. In a complex setting, distributed leadership has been found to increase the capacity for learning (Champagne *et al.*, 2014) and champion teams promote change more effectively than lone champions (Kirchner *et al.*, 2012).

With regard to networks and communication, differences were seen between high and low-performing GACOs. Cohesion and collaboration in the GACO team were more prominent in high-performing cases, and were reflected in regular interaction and enhanced communication, as seen between the nurse and clerk in one GACO who collaborated to solve problems and respond to patients’ needs. The role of communication is well established in implementation science (Damschroder and Lowery, 2013; Greenhalgh *et al.*, 2004). Our results concur with Damschroder and Lowery (2013) findings that cases of effective implementation are more likely to exhibit better working relationships, ongoing communication flows and higher-functioning teams. Gilmer *et al.* (2013) likewise emphasized the importance of language and communication channels in the implementation of a client-centered service for persons with mental illness.

One notable finding in the inner setting relates to how staff dealt with resource constraints, which is a common challenge in the implementation literature. While all sites faced resource challenges, only high-performing GACOs were able to develop creative strategies to optimize existing resources; low-performing sites displayed inertia and were unable to overcome resource barriers. Our results offer useful insights not only around factors that influence implementation, but also into strategies that key stakeholders (GACO staff) put in place to overcome implementation challenges. Similar strategies have been adopted when implementing advanced access, an innovation to improve timely access to primary care (True *et al.*, 2013), showing that stakeholders are not passive recipients of innovation (Greenhalgh *et al.*, 2004), but rather active players in the change process who interact creatively with an innovation and react to challenges with internally developed solutions (Greenhalgh *et al.*, 2004). Nevertheless, decision makers should know that resource availability (human, time, financial resources) is an essential condition to enhance implementation success and sustain innovations, as shown in studies similar to ours (Greenhalgh *et al.*, 2004; Varsi and Ekstedt, 2015; Gilmer *et al.*, 2013), and in many other studies related to implementation of healthcare innovations in general (Fleuren *et al.*, 2004, 2014; Greenhalgh *et al.*, 2004; Kilbourne *et al.*, 2007).

With regard to the innovation (GACO) characteristics, our results indicate that high-performing GACOs were more innovative in embracing complexity than low-performing GACOs, and adapted the innovation to their local settings and the needs of patients on the waiting list. GACOs required continuous adaptation by staff. Indeed, adaptation is likely to occur when complex innovations unfold in real-world context (Pérez *et al.*, 2016) involving multiple organizational levels: the GACO is implemented within the HSSC where it is physically located, and in between all the primary care structures of the LHN around the HSSC.

Flexibility in adaptation is described in the literature as being closer to a user-based or bottom-up approach (Pérez *et al.*, 2016). This might be explained in part by the fact that the province has mandated implementation at a local level without developing detailed national guidelines (Breton, Smithman, Brousselle, Loignon, Touati, Dubois, Nour, Boivin, Berbiche and Roberge, 2017) to which implementers/users must strictly adhere, and to different leadership responses in the GACOs studied. Regardless of why flexibility exists, the ability to adapt an innovation increases its acceptability among local users (Rogers, 2003). In our study, adaptability contributed to achieving GACO outcomes (e.g. prioritizing vulnerable populations, etc.) as some GACO staff were able to challenge the existing power relationships with family physicians. Researchers acknowledge that a less prescriptive state-mandated reform encourages creativity and may provide an opening for innovation by institutional entrepreneurs who adopt a proactive strategy to influence the change process (Breton, Lamothe and Denis, 2014).

The implementation, dissemination and sustainability of complex organizational models such as GACOs is likely to require a balance between strategic (top-down) directives and the tacit knowledge of local stakeholders (patients served by the GACO, healthcare providers), who contribute empirically grounded knowledge of the local context and their own lived experience (Farmer *et al.*, 2018). The strategies used to adapt GACOs in the cases studied here could be tested on other sites and refined through the participation of diverse healthcare providers and GACO beneficiaries. Such a process could lead to co-produced and contextualized national guidelines that could improve the quality of delivery of GACOs, and better equip them to achieve the desired outcomes. Conducting implementation research on the GACO innovation over time and across settings, such as recommended by Chambers and Norton (2016), could help to generate the information needed to continue refinements and meet the needs of broader and more diverse populations.

It is important to note that in 2016, the innovation underwent major changes: centralization at the Quebec health insurance board of Quebec (the RAMQ) and management at provincial level; and prioritization according to five new categories reflecting urgency and health needs (Breton, Green, Kreindler, Sutherland, Jbilou, Wong, Shaw, Crooks, Contandriopoulos, Smithman and Brousselle, 2017). The changes do not, however, reduce the value of our results that remain informative: adaptation is still needed to overcome external factors shown to be consistent across four GACO sites (e.g. patients who miss appointments, cultural/language barriers) (Chambers and Norton, 2016) and to meet the population needs.

Despite the numerous studies examining models that similarly aim to improve timely access to healthcare in different countries (England, USA, Canada), very few have identified and explained factors that differentiate high from lower-performing sites in the implementation of such innovations. Studies that have attempted to address this relationship (Pope *et al.*, 2008; Breton *et al.*, 2016) show that variation with respect to implementation stems mainly from organization-level factors (leadership strategy, availability of human resources: nurses, physicians) (Abou Malham *et al.*, 2017) and from factors such as misunderstanding of the innovation (Pope *et al.*, 2008).

Given that there are no comparative studies on implementation of CWLs in primary healthcare, and very few on similar models, it would be worth exploring additional sites to expand knowledge regarding CWLs design and identify the most influential factors involved in variation between high- and low-performing sites.

### Strength and limitations

The strength of this study lies, first, in its coverage of almost all GACO staff within the four sites (apart from Site 2), and interviews with family physicians, who are knowledgeable informants directly involved in GACOs implementation and/or impacted by the GACOs. Second, the researchers who coded and analyzed data were blinded to the status of implementation in the four GACOs, which helped reduce bias in qualitative findings and ensure trustworthiness. Double coding was performed using an iterative process, which also helped to increase credibility. Analyzing the results through the CFIR helped synthetize results, will facilitate future comparison of findings across other similar studies adopting the same methodology.

A few limitations to the current study should be mentioned. First, the number of participants at Site 2 was less than the number of the other three sites and may have yielded a less rich picture. Additional results may have been captured if the GACO nurse had agreed to be interviewed.

Second, we selected four sites among 94, given our objective was not to produce statistically generalizable results, but rather a rich contextualized understanding of each GACO. The final limitation of this study relates to the absence of patients’ experience with the GACO. Future research should include interviews with patients who were attached with a family physician through these CWLs, and with those who remain on the waiting list.

We also point out that we have presented in detail only 5 among a possible 39 distinguishing constructs. We do not consider this problematic given that use of a limited number of constructs has been recommended by the designers of the framework (Damschroder and Lowery, 2013) and others (Breimaier *et al.*, 2015) for implementation analysis aiming to differentiate between high and low implementation effectiveness. Moreover, we did not use the constructs to guide data collection; our semi-structured interview guide was based on the large domains of the CFIR.

## Conclusion

This study provides the first in-depth analysis of CWLs implementation. Findings can be used to develop strategies to overcome barriers to implementation, better manage wait lists, and improve performance. Ultimately, they could contribute to reducing inequities in access to a family physician, and in health outcomes, notably for vulnerable populations and those with complex physical and/or mental healthcare needs. Findings are also relevant for decision makers responsible for designing complex innovations whose decisions shape the development, implementation and scale-up of CWLs. They may also more generally inform the dissemination efforts of similar complex organizational models in different contexts. When implementing this innovation in similar real-world healthcare delivery contexts, and when redesigning implementation strategies, greater consideration should be given to the combination of organization-level factors (leadership engagement, resource availability, networks and communication), intervention characteristics (adaptability) and the process domain (engagement) identified as factors important to achieving implementation outcomes. Moreover, a mandated innovation that is simultaneously top-down and less prescriptive creates a good opportunity for stakeholders in the field to identify practical ways to bring about change.

## Figures and Tables

**Table I:** Relative performance of the four selected GACOs (2013–2014)

	Lower performing cases		Higher performing cases	
Case name	Low-Case 1	Low-Case 2	High-Case 3	High-Case 4
Region	Montérégie	Montréal	Montérégie	Montréal
*Implementation process outcomes*				
New requests for an FP^a^	Low	Low	High	Average
Change in the number of patients waiting for an FP^b^	Average	Low	Average	High
Patients attached to an FP^c^	Low	Low	High	High
Vulnerable patients attached to an FP^d^	Average	Average	Average	Average

**Notes:** FP, family physician; GACO, Guichets d’accès aux clientèles orphelines. ^a^New request for an FP: includes every new request made in 2013–2014. It reflects population and health professional knowledge of the GACOs’ existence, as well as how easy it is to submit a request for an FP to the GACO. Larger numbers of new requests were considered to indicate better GACO performance; ^b^change in the number of patients waiting for an FP on the GACO: captures whether the number of patients waiting for an FP in the GACO increased or decreased during 2013–2014. Greater decrease in number of patients on the list was considered to reflect better GACO performance, since it indicated that more patients were attached to an FP than there were new requests for an FP during the year; ^c^patients attached to an FP through GACOs: measures the number of patients who were attached to an FP through GACOs during the year 2013–2014. Larger numbers of patients being attached to an FP during the year was considered to indicate better GACO performance; ^d^vulnerable patients attached to an FP through GACOs: measures the proportion of patients attached to an FP through GACOs during the year who were identified as vulnerable (with at least one of the 19 vulnerability codes). GACOs that had attached a larger proportion of vulnerable patients were considered to have better performance, in terms of success in prioritizing patients with greater health needs

**Table II:** Description of the CFIR rating rules

Rating	Description (valence and strength)
−2	Construct has a negative influence, i.e.: impeding influence in the organization, in work processes, and/or in implementation efforts The majority of interviewees (at least two) describe explicit/concrete examples of how key or all aspects (or their absence) of a construct manifest in a negative way
−1	Construct has a negative influence on implementation efforts No explicit examples provided; is mentioned only in passing
0	Construct appears to have a neutral effect Is only mentioned generically (purely descriptive) No evidence of positive or negative influence Contradiction among interviewees Presence of positive and negative influences at different levels in the organization that balance each other out; and/or different aspects of the construct have positive influence while others have negative influence which make the overall effect neutral
+1	Construct has a positive influence, i.e.: facilitates implementation efforts Is mentioned only in passing (no explicit examples)
+2	Construct has a positive influence, i.e.: facilitating influence in work processes, and/or a facilitating influence in implementation efforts The majority of interviewees (at least two) describe explicit/concrete examples of how key or all aspects of a construct manifest in a positive way

**Note:** Adapted from Damschroder and Lowery (2013)

**Table III:** Matrix presenting the ratings for the outer setting constructs based on the CFIR framework by level of GACO performance (or by level of implementation outcome)

	Low performers		High performers		
CFIR domains and constructs	GACO 1	GACO 2	GACO 3	GACO 4	Distinguishing constructs
*Outer setting*					
External policies and	0	0	0	0	X
Incentives	0	Missing	0	0	X
Patients’ needs and resources	−2	−2	−2	−2	X

**Note:** X: Constructs do not distinguish between low and high performers

**Table IV:** Matrix presenting the ratings for the inner setting constructs based on the CFIR framework by level of GACO performance (or by level of implementation outcome)

	Low performers		High performers		
Domain/constructs	GACO 1	GACO 2	GACO 3	GACO 4	Distinguishing constructs
*Inner setting*					
Networks and communication	−1	−1	+2	+2	^a^
Within HSSC	+1	0	+2	+2	
Within LHN	−2	−1	+2	+2	
Leadership Engagement	0	0	+2	+2	^a^
Available resources and strategies to overcome this barrier	−2	−2	+2	+2	^a^
Tension for change	−1	−1	−1	Missing	X
Compatibility	+1	Missing	−1	Missing	X
Relative priority	Missing	−1	−1	−1	X
Goals and feedback	+1	0	+1	+1	X
Access to knowledge and information	−1	−1	Missing	−1	X

**Notes:** X: Constructs do not distinguish between low and high performers. ^a^Constructs strongly distinguish between low and high performers

**Table V:** Matrix presenting the ratings for the innovation characteristics constructs based on the CFIR framework by level of GACO performance (or by level of implementation outcome)

	Low performers		High performers		
	GACO 1	GACO2	GACO3	GACO4	Distinguishing constructs
*Innovation characteristics*					
Adaptability	0	−1	+2	+2	^a^
Registration	0	0	+2	+2	
Prioritization	+1	0	+2	+2	
Matching	−1	−1	+2	+1	
Relative advantage	0	0	0	+1	X
Complexity	−1	−2	−2	−2	X

**Notes:** X: Constructs do not distinguish between low and high performers. ^a^Constructs strongly distinguish between low and high performers

**Table VI:** Matrix presenting the ratings assigned to the individual characteristics constructs based on the CFIR framework by level of GACO performance (by level of implementation outcome)

	Low performers		High performers		
	GACO 1	GACO2	GACO3	GACO4	Distinguishing constructs
*Individual characteristics*					
Knowledge and beliefs	−1	−1	−1	0	X
Personal attributes	0	0	0	0	X

**Note:** X: Constructs do not distinguish between low and high performers

**Table VII:** Matrix presenting the ratings for the process domain constructs based on the CFIR framework by level of GACO performance (by level of implementation outcome)

	Low performers		High performers		
	GACO 1	GACO2	GACO3	GACO4	Distinguishing constructs
*Process*					
Engaging	−2	−1	+2	+1	^a^

**Note:** ^a^Constructs strongly distinguish between low and high performers

**Table VIII:** Constructs of the inner setting according to the CFIR framework distinguishing high-performing cases from lower ones

	Low-performing cases		High-performing cases	
	Case 1	Case 2	Case 3	Case 4
*Inner setting*				
1.1 Networks and communication within services of the HSSC	Communication/meetings Between GACO personnel Informal meeting to address common work issues between clerk and GACO nurse Trying to improve work procedures (e.g. clerk work)	Communication/meetings Between GACO personnel Some meetings between GACO staff Still trying to improve work procedures (e.g. clerk work)	Communication/meetings Between GACO personnel Good collaborative practice and communication within GACO staff and with the medical coordinator: to take decisions about strategies and about special cases; to ensure a work continuity: e.g. using their initials to leave comments in the system Formal communication strategy developed through a computer-based system online, to perform a quick pre-prioritization process for patients with special needs and reduce delays	Communication/meetings Between GACO personnel Good collaborative practice and communication between GACO staff to accelerate registration and evaluation (e.g. monthly meetings) and with medical coordinator Communication system instituted either via formal or informal procedures: using staff initials and developing symbol systems and codes between nurse and clerk to identify priority patients quickly and enhance their access to FP
	Between GACO personnel and HSSC personnel Informal communication mechanisms between GACO personnel and other HSSC personnel to facilitate the process of discharging a patient	Between GACO personnel and HSSC personnel Collaboration on some occasions with HSSC personnel	Between GACO personnel and HSSC personnel Communication highly developed: several meetings within the HSSC	Between GACO personnel and other HSSC personnel Tight link between GACO with other services of the HSSC to give care to patients while waiting on GACO
1.2 Networks and communication in between the services of the LHN	Between GACO staff and clinics, health professionals Poor communication and collaboration GACO does not work with all clinics of the LHN	Between GACO staff and clinic, health professionals Poor/very little communication, collaboration FP working in clinics not being adequately informed about patients’ waiting lists, changes in GACO’s procedures Some contacts between GACO nurse and FP: facilitates referral of some cases, patients’ transfer in case of FP’s retirement	Between GACO staff and clinic, health professionals Inter professional communication, collaboration well developed Sharing a medical file on patients referred from GACO: facilitate their follow up with patient, increase FP will to participate in GACO Meeting with new FP before starting their practice and referring them to GACO to ask for new patients; with several FP to ask them to take patients from GACO	Between GACO staff and clinic, health professionals Inter professional communication, collaboration well developed Good contact between GACO clerk, nurse and some clinics facilitates patients care in case of emergency Tight link between GACO and other services of the HSSC to provide care to patients while they are on the waiting list Sharing a complete form on patients with health assessment (evaluation tool) with clinics Meetings and discussions by GACO staff with clinics to resolve communication’s difficulty Sometimes poor communication between GACO staff–clinics–FP: (e.g. follow up by GACO staff with clinics to know if patients have been refused) Collaboration of GACO nurse with other health professionals: nurse who registers homeless patients collaborates/meets the health professional to make the GACO well-known, to enhance taking care of homeless people
	Between clinics Most clinics of territory function solitarily thus hampering their participation in GACO Most clinics of the LHN do not collaborate, no networking between the different LHN	Between clinics Links not mentioned	Between clinics Well-established links between clinics and services Communication with RDGM, FMG, HSSC, all FP: important to maintain for GACO Good link between all the services of the different territories: meeting every month at regional level and several meetings within the services of HSSC	Between clinics Tight link between services of the HSSC to provide care to patients waiting on the GACO
*Readiness for implementation*				
2. Leadership engagement	No specific champion mentioned, unknown by some FP Medical coordinator: not very aggressive in his interventions, to invite his colleagues (FP) to participate in GACO Traditional role fulfilled by medical coordinator: providing support when needed, dealing with problematic FP	Involvement of a champion FP Active medical coordinator: in managing patients’ requests, solving problems, taking many vulnerable patients Absence of medical coordinator during a period of time: limited capacity of manager fulfilling this role to recruit FP Procedure to improve GACO’s functioning not done, is still on hold due to the leader’s departure	Involvement of a champion FP Medical coordinator highly involved in GACO implementation: played a major role in solving problems, encouraging FP to attach patients, in managing patients’ requests and attaching many vulnerable patients Proactive leader: creative initiatives to offer alternative health services	Involvement of two emergent champions (not formally appointed) One young FP highly involved (attached 400 vulnerable patients-babies from GACO/year; very well known by others) Nurse: very proactive, keen on reducing long waiting times for patients, took the responsibility to create adaptive strategies to leverage resources Lack of involvement of medical coordinator, of playing active role in implementation No leadership exerted by him: described as not motivated to explain the GACO’s functioning to other FP Unknown by some FP
3. Available resources	Lack of human resources, financial resources No extra support from the HSSC in GACO implementation Presence of one nurse in GACO who has to perform many tasks, 3 working days instead of 5 due to financial budgets cut Clerk will be working alone because nurse is leaving Lack of resources, support in clinics for FP to take more patients from GACO No strategy adopted to counteract this barrier	Lack of human, financial resources, turnover of staff, technology difficulty Lack of staff, FP on the territory to participate in GACO, to answer the patients’ needs Important staff turnover: lack of clerk’s stability leads to a delay Not enough funds for recruiting more human resources Technologic resources: SIGACO was not linked to Quebec Health Insurance Board (RAMQ) No strategy adopted to counteract this barrier	Lack of human resources, lack of time, turnover of staff, Technology difficulty Not enough human resources to manage registration by phone, due to many requests Not enough FP to take patients from GACO, to answer to all patients’ requests Not enough space also Access to Quebec Health Insurance Board (RAMQ) to check patient attachment is not easy for GACO staff Adoption of strategy to optimize resources Pre-prioritization done by computer to overcome lack of human resources Mobilizing a nurse clinician to help GACO’s staff (to prioritize patients, check their status in the medical insurance database) Suggestions made: double the staff to be able to run the GACO	Lack of human, financial resources, lack of time, turnover of staff, technology difficulty Not enough resources to manage the numerous requests for GACO Not enough FP to respond to demand (many requests) Funds constrains to hire several nurses Adoption of strategy to optimize resources Optimal utilization of resources: full-time clerk and several nurses not dedicated to prioritization but will help when they have free time in their agenda Training many nurses for evaluation in GACO during free time on their schedule in order not to depend on only one person Suggestions made: dedicate one nurse to answer requests through one phone line

**Notes:** FP, family physician; GACO, Guichets d’Accès aux Clienteles Orphelines; FMG, Family Medicine Group; HSSC, Health Social Services Centers; LHN, Local Health Network; QFGP, Quebec Federation of General Practitioners; RAMQ, Régie d’Assurance Maladie du Quebec (Quebec Health Insurance Board); RDGM, Regional Department of General Medicine; SIGACO, database
